# Effects of PB-EPCs on Homing Ability of Rabbit BMSCs via Endogenous SDF-1 and MCP-1

**DOI:** 10.1371/journal.pone.0145044

**Published:** 2015-12-14

**Authors:** Hanxiao Wei, Xian Zhao, Ruihong Yuan, Xiaoming Dai, Yisong Li, Liu Liu

**Affiliations:** Department of Plastic Surgery, the First Affiliated Hospital of Kunming Medical University, Kunming, Yunnan Province, PR of China; University of Connecticut Health Center, UNITED STATES

## Abstract

Traumas, infections, tumors, and some congenital malformations can lead to bone defects or even bone loss. The goal of the present study was to investigate whether inclusion of endothelial progenitor cells derived from peripheral blood (PB–EPCs) in cell-seeded partially deproteinized bone (PDPB) implants would stimulate recruitment of systemically injected bone marrow stromal cells (BMSCs) to the implant. Methods: BMSCs were injected intravenously with lentiviral expression vector expressing enhanced green fluorescent protein (eGFP) for tracing. Recruitment of eGFP-positive BMSCs was tested for the following implant configurations: 1) seeded with both BMSC and PB-EPC, 2) BMSC alone, 3) PB-EPC alone, and 4) unseeded PDPB. Protein and mRNA levels of endogenous stromal-derived factor-1 (SDF-1) and its receptor CXCR4, as well as monocyte chemotactic protein-1 (MCP-1) and its receptor CCR2, were evaluated on the 8th week. Immunohistochemical staining was performed to determine eGFP-positive areas at the defective sites. Masson’s trichrome staining was conducted to observe the distribution of collagen deposition and evaluate the extent of osteogenesis. Results: The mRNA and protein levels of SDF-1 and CXCR4 in the co-culture group were higher than those in other groups (*p < 0*.*05*) 8 weeks after the surgery. MCP-1 mRNA level in the co-culture group was also higher than that in the other groups (*p* < 0.05). Immunohistochemical assays revealed that the area covered by eGFP-positive cells was larger in the co-culture group than in the other groups (*p* < 0.05) after 4 weeks. Masson’s trichrome staining revealed better osteogenic potential of the co-culture group compared to the other groups (*p* < 0.05). Conclusion: These experiments demonstrate an association between PB-EPC and BMSC recruitment mediated by the SDF-1/CXCR4 axis that can enhance repair of bone defects.

## Introduction

Bone defects and morphological abnormality caused by trauma and congenital malformation are common clinical problems. Studies have shown that autologous bone graft is likely to cause donor site defects. Therefore, developing methods for combining cytology and materiology to construct tissue-engineered bone is an important goal in regenerative medicine.

Bone marrow stromal cells (BMSCs) are popular stem cells in tissue engineering and regenerative medicine. Osteoblasts rapidly decrease in the defective area when bone defects occur, limiting the potential for recovery from bone damage. Transplantation of BMSCs can increase the quantity of osteoblasts and accelerate bone repair. Previous studies focused mainly on promoting speedy seed cell proliferation and rapid vascularization [1–2]. However, when implanting tissue-engineered bones into the body, attention should be paid to the transformation of exogenous stem cells and the potential of intrinsic mesenchymal stem cells to accelerate bone repair. Repair of tissue-engineered bones can be accelerated if intrinsic stem cells move toward the defects while promoting proliferation and osteogenesis of seed cells. Thus, promotion of stem cell homing is a key challenge in the development of regenerative medicine, from construction of 3D structures to clinical applications.

Endothelial progenitor cells (EPCs) were first identified in adult human peripheral blood [3]. Considering the excellent self-proliferation abilities and pluripotency of EPCs [4, 5], the use of EPCs instead of vascular endothelial cells as adult stem cells has increasingly become the focus of research in recent years. In tissue-engineered bone construction, EPCs are often used in rapid vascularization of bone tissues [6–9] and formation of new bones [10, 11]. Further understanding of EPC functions and characteristics can elucidate the mechanism underlying EPC-based bone repair. Moreover, EPCs can promote earlier vascularization and osteogenesis of tissue-engineered bones, as well as provide nutritional support to seed cells co-cultured with them through paracrine and adhesive capacities [12, 13]. EPCs release VEGF-A through paracrine signaling, which can also promote MSC proliferation [14, 15]. Additionally, EPCs release bone morphogenetic protein (BMP), which can promote MSCs expressing osteopontin and osteocalcin [16, 17].

Many recent studies have reported on the functions of chemotactic axes to treat diseases. Stromal cell-derived factor-1 (SDF-1), which is highly conserved between species, is a CXC chemokine protein generated in MSCs and functions by binding to CXCR4 [18]. Binding of SDF-1 and its receptor CXCR4 leads to activation of the SDF-1/CXCR4 axis, which is important in the recruitment of BMSCs and directed migration. Kitaori T showed that SDF-1/CXCR4 signaling is critical for the recruitment of MSCs to a fracture site during skeletal repair in mice [19]. Moreover, SDF-1 therapy has been shown to cause increased cell migration, neovascularization, and tissue repair in ischemic cardiovascular disease [20]. Besides the SDF-1/CXCR4 axis, the MCP-1/CCR2 axis is also involved in chemotaxis. Belema et al. [21] used DNA chip technology and in vitro migration assays and found that presence of CCR2 was necessary for the movement of BMSCs to sites of cardiac ischemia. Furthermore, MCP-1 was also found to recognize CCR2 and trigger BMSC polarization and cytoskeletal protein rearrangement, leading to migration.

Based on these information, we hypothesized that EPCs may promote mobilization of BMSCs through the SDF-1/CXCR4 and MCP-1/CCR2 axes related to homing and thereby facilitate construction of bone defects. In this study, we established a rabbit bone defect model and co-cultured partially deproteinized bone (PDPB) with BP–EPCs and BMSCs to construct a tissue-engineered bone. The homing behavior of BMSCs was monitored using BMSCs transduced with lentivirus carrying enhanced green fluorescent protein (eGFP). Expression of components of the SDF-1/CXCR4 and MCP-1/CCR2 axes related to stem cell homing was analyzed. We found an association between PB-EPC and area of BMSC that is mediated through endogenous SDF-1 and MCP-1 to repair bone defects.

## Materials and Methods

### Ethics statement

This experiment was approved by the Institutional Animal Care and Use Committee of Kunming Medical University. All aspects of the animal experiments were conducted in accordance with the approved protocol. Animals were anesthetized with 10% chloral hydrate at a dose of 2 mL/kg, and all efforts were made to minimize their suffering.

### Materials

MSCM (Sciencell), EGM-2 BulletKit (Lonza), L-DMEM (Gibco), PBS (Life), EDTA (Cxbio), monoclonal antibodies specific for rabbit CD29, CD34, CD45 (BD Bioscience, San Diego, CA), eGFP expression plasmid (GuangZhou FuNeng), lentivirus kit (Gene Copoeia), low-density lipoprotein acetylated DiI complex (Invitrogen), FITC-labeled Ulex europaeus agglutinin I (Sigma), Matrigel (BD Biosciences), SDF-1/MCP-1 enzyme-linked immunosorbent assay (ELISA) kits (Cloud-clone Corp), DEPC (AMRESCO), Trizol (MRC, TR118), Revert Aid TM First Strand cDNA Synthesis Kit (Fermentas), SYBR Green Master Mix (Fermentas), CXCR4, CCR2 primary antibody (Santa Cruz), PVDF membranes (BioRad), eGFP primary antibody (Millipore), eGFP secondary antibody kit (ZhongShan JinQiao pv-9000), and Masson’s trichrome staining kit (Sigma) were the materials used in this experiment.

### Methods

#### Isolation of rabbit BMSCs and PB–EPCs

Rabbit BMSCs: Anterior superior iliac spine was sterilized and draped conventionally and then exposed under aseptic condition. Puncture needle was slowly injected 1 cm below the anterior superior iliac spine to extract bone marrow aspirate. The extracted 5 mL of bone marrow aspirate was slowly transferred into a centrifugal tube along its wall and laid onto Percoll solution at a ratio of 1:1. Buffy coat was collected after centrifugation. BMSC complete medium with serum and penicillin–streptomycin solution was added to the buffy coat and mixed well. After dispersing the cell mass, the mixture was transferred into a plastic cell culture flask and then cultured in a 5% CO_2_ incubator at 37°C. The medium was replaced after 48 h, and all suspended cells were removed. The medium was then replaced every 24 h. Changes in cell morphology were observed under an inverted phase-contrast microscope.

Rabbit PB–EPCs: Approximately 5 mL of venous blood sampled from the edge of the rabbit’s ear was mixed with Percoll solution for centrifugation. Cells were separated through centrifugation, cultured in EGM-2 BulletKit endothelial cell growth medium, and then inoculated on a fibronectin-coated plate. The plate was placed in 5% CO_2_ incubator at 37°C, and the medium was replaced every 24 h. Changes in cell morphology were observed under an inverted phase-contrast microscope.

#### Flow cytometry

When the density of BMSCs reached 80%, 2 mL of 0.25% pancreatic enzyme solution containing EDTA was added to the culture. Culture medium was added to stop the digestion after the cells detached from the plate. Cell mass in the medium was dispersed by blowing to obtain a cell suspension. PBS was added to the cell suspension to adjust the cell concentration to 106/mL; 20 μL of the corresponding monoclonal antibodies specific for rabbit CD29, CD34, CD45 were added to a flow cytometry tube and mixed with 100 μL of cell suspension. Cells incubated without antibody served as control. The mixture was incubated without light for 30 min at 4°C. Afterward, unbound antibodies were washed off.

#### Characterization of EPCs

The cultured cells were confirmed to be EPCs by detecting the presence of both low-density lipoprotein acetylated DiI complex (acLDL) and FITC-labeled Ulex europaeus agglutinin I (UEA- I), as well as forming 2D networks on Matrigel, which were commonly referred to as EPC characteristics, as previously demonstrated [22, 23]. Briefly, cells were incubated with 2.5 μg/mL acLDL at 37°C for 3 h. The cells were then fixed with 2% paraformaldehyde for 10 min. After washing, the cells were counterstained with 10 μg/mL FITC-labeled UEA-1 for 1 h at 37°C. Stained cells were viewed using a fluorescent microscope. Matrigel complex was diluted at 1:1 ratio with EGM-2 on ice after plating the mixture for 3 h. The cells were seeded on a 24-well plate pre-coated with Matrigel matrix. Tubes were observed under an inverted light microscope.

#### Preparation of BMSCs expressing eGFP

BMSCs cultured to the third generation with a density of 1 × 10^5^/mL were inoculated onto six-well culture plates. When the extent of cell confluence reached approximately 80%, 3 mL of culture medium and 1 mL of recombinant eGFP-carrying lentivirus (1.39 × 10^8^ copies/mL) were added to the culture. The culture flask was shaken slowly and then placed in an incubator at 37°C. For screening, BMSCs were transferred to DMEM/F-12 culture medium containing 0.5 μg/mL puromycin. The inserted exogenous plasmid was resistant to puromycin. Hence, we used puromycin to screen the stable expression of eGFP in BMSCs. After 3 weeks, cell growth and expression of GFP were observed. Transduced BMSCs, which were passaged thrice, were injected intravenously into the rabbit bone defect model.

#### SDF-1 and MCP-1 protein expression of BMSCs expressing eGFP in ex vivo cultures

The transduced BMSCs were placed in a six-well plate with a density of 10^5^/mL. Cells were analyzed on days 3, 7, and 14. Culture medium was changed 48 h before the assay, and supernatant was collected from the cultured cells. Protein content was determined using SDF-1 and MCP-1 ELISA kits in accordance with the supplier’s instructions, with a minimum detectable concentration of 18 pg/mL. Absorbance was determined at 450 nm. SDF-1 and MCP-1 contents were calculated using the absorbance values of the reference standard and samples. Each experiment was repeated thrice.

#### Formation of biological bone from a combination of partially deproteinized bone (PDPB) and seed cells

A pig vertebra obtained from the market was crushed to make bone fragments. These bone fragments were repeatedly washed with distilled water and immersed in ethanol for 24 h. The bone fragments were then immersed in distilled water for 30 min and in acetone for 24 h, with the solution pH adjusted between 7.0 and 7.2. Bones were dried in a drying oven to obtain PDPB after immersion. PDPBs were ground into bone fragments with sizes of 1.2 cm × 0.4 cm × 0.3 cm, which were washed with normal saline by using ultrasonic cleaner, and then dried and sterilized by radiation for later use. PDPBs were randomly divided into four groups: group 1, PDPBs cultured with BMSCs and PB–EPCs (10^6^/mL total at 1:1 ratio, each type cell 5╳10^5^/mL); group 2, PDPBs cultured with BMSCs alone (10^6^/mL); group 3, PDPBs cultured with PB–EPCs alone (10^6^/mL); and group 4, PDPBs cultured alone. Complexes were cultured for 7 days with 4 mL of complete medium. The culture medium of all groups were replaced daily. The 7-day-old complexes were fixed with 30 g/L pentodialdehyde, 1% osmium acid post-fixation, ethanol desiccation, and isopropyl acetate replacement for observation under a scanning electron microscope.

#### Establishment of rabbit radial defect model and grouping

Healthy New Zealand white rabbits aged 4 weeks and weighing 800±10 g received intraperitoneal anesthesia via injection of 10% chloral hydrate at a dosage of 2 mL/kg. After routine sterilization and draping, 12 mm long defects were made in the middle radius of bilateral forearms under aseptic condition through skin incisions. The periosteum was then removed and a 12 mm critically sized defect was osteotomized using a bone-grinding instrument. A total of 48 rabbits were randomly divided into four groups (*n* = 12 each) to receive the following implants: Co-culture group, in which animals were implanted with PDPBs seeded with both BMSC and PB-EPC (i.e., injected BMSC would augment the number of seeded BMSC); BMSC group, in which animals were implanted with PDPBs seeded with BMSC alone (i.e., implanted BMSC would recruit injected BMSC); EPC group, in which animals were implanted with PDPBs seeded with EPC alone (i.e., implanted PB-EPC would recruit injected BMSC); and Unseeded group, in which animals implanted with PDPBs that were unseeded (i.e., baseline control to account for injected BMSC recruitment to the wound site). PDPBs of the four groups were precisely implanted in the defects using a stainless plate fixed with screws on the proximal and distal ends of the radius. The wounds were then sutured layer by layer. Rabbits were reared in separate cages under the same condition and injected with gentamicin sulfate at 2 × 10^4^ units per day to prevent infection. Rabbits that developed fast heartbeat and breathing received 10% chloral hydrate at a dose of 0.5 mL/kg. Rabbits were monitored daily for any complication or abnormal behavior following surgery. After 48 h, rabbits from each group were injected through the caudal vein with BMSC suspension with stable expression of eGFP at a concentration of 5 × 10^6^/mL. No cases of infection, fracture, or death occurred during the course of the experiment.

#### Quantitative real-time PCR assay

PDPB-embedded rabbit bone tissues were harvested after the animals were sacrificed through an overdose of chloral hydrate at 8 weeks after surgery. Samples were ground and treated with Trizol to extract the total RNA of cells, as recommended by the manufacturer. Afterward, cDNA was immediately synthesized. Second-strand synthesis and amplification were performed by mixing 1 μL of cDNA, 2 μL of PrimerScript RT Master Mix, 1 μL of PCR forward primer, 1 μL of PCR reverse primer, and pure water to obtain a total volume of 10 μL. The following PCR conditions were used: 95°C for 5 min followed by 40 cycles of 95°C for 15 s, 57°C for 15 s, and 72°C for 30 s. The Ct value was recorded, and GAPDH was used as an internal reference. All reactions were performed in triplicate. Relative gene expression was calculated using the 2^−ΔΔCt^ method.

#### ELISA

The SDF-1 and MCP-1 levels of each cell group were measured using ELISA. A specific ELISA kit was employed with a minimum detectable concentration of 18 pg/mL. Briefly, serum samples were collected at 8 weeks postoperatively. Standard wells, sample wells, and blank wells were diluted as instructed by the corresponding suppliers. Absorption at 450 nm was determined using a microplate reader. The SDF-1 and MCP-1 levels in each serum sample group were calculated using the absorbance values of the reference standard and samples. Each experiment was repeated thrice.

#### Western blot

Subsequently, we investigated whether SDF-1 receptor CXCR4 and MCP-1 receptor CCR2 were involved in the homing ability of BMSCs. The material-embedded rabbit bone tissues were harvested after the animals were sacrificed through an overdose of chloral hydrate at 8 weeks after surgery. Protein lysates were obtained in a RIPA lysis buffer with protease inhibitors and protein concentrations determined using Bradford assay (BioRad). Approximately 50 μg of protein was separated on precast 10% SDS-PAGE denaturing gels, which were then transferred to PVDF membranes. Western blot analysis was performed with anti-rabbit CXCR4 and anti-rabbit CCR2 primary antibodies, as well as with relevant secondary antibodies by using standard techniques [24]. All values were averaged at least thrice and normalized to the constitutive actin expression.

#### Immunohistochemistry

The material-embedded rabbit bone tissues were harvested at 2, 4, and 8 weeks after surgery and then fixed in 4% paraformaldehyde. A hard tissue slicer was used to cut the tissues into 4 μm-thick sections. Briefly, deparaffinized sections were washed with PBS. Subsequently, eGFP primary antibodies (1:100 dilution) were incubated overnight at 4°C. Polymer helper- and poly peroxidase-tagged anti-rabbit IgG were then dropped to the sections, which were later incubated at 37°C and developed with DAB solution. The sections were routinely sealed and examined under a microscope. Three sections were randomly selected for each sample, and five visual fields were randomly selected for each section. The positively stained area of eGFP was calculated using Image-ProPlus 6.0 image analysis system.

#### Histological analyses

Sections were collected via immunohistochemistry at 2, 4, and 8 weeks after surgery. Sections of each group received Masson’s trichrome staining to study the collagen deposition and evaluate the new bone formation of the samples. The distribution of collagen deposition was detected as blue color by using Masson’s trichrome staining kit in accordance with the manufacturer’s instructions. The region of collagen deposition in the samples was calculated using Image J 4.5 version software. Three selected sections from randomly chosen high-power fields (HPFs) were quantified and analyzed. The number of pixels obtained from the three HPFs was summed.

### Statistical analysis

Data were analyzed using SPSS 21.0 statistical software. All data were presented as average ± standard deviation (x ± s). One-way ANOVA was conducted for intergroup comparison. P < 0.05 was considered statistically significant.

## Results

### Cell morphology and identification

**Fig 1:**
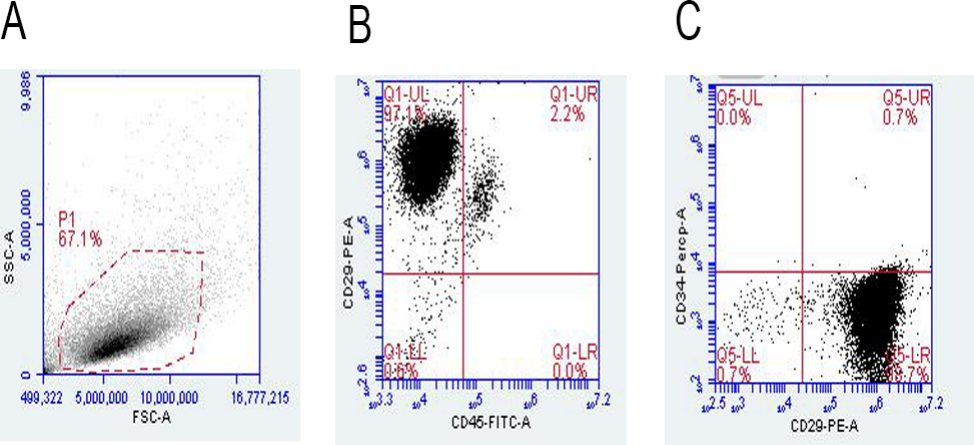
BMSC characterization. A: control group. B: BMSCs were positive for CD29 and negative for CD45. C: BMSCs were positive for CD29 and negative for CD34.

**Fig 2:**
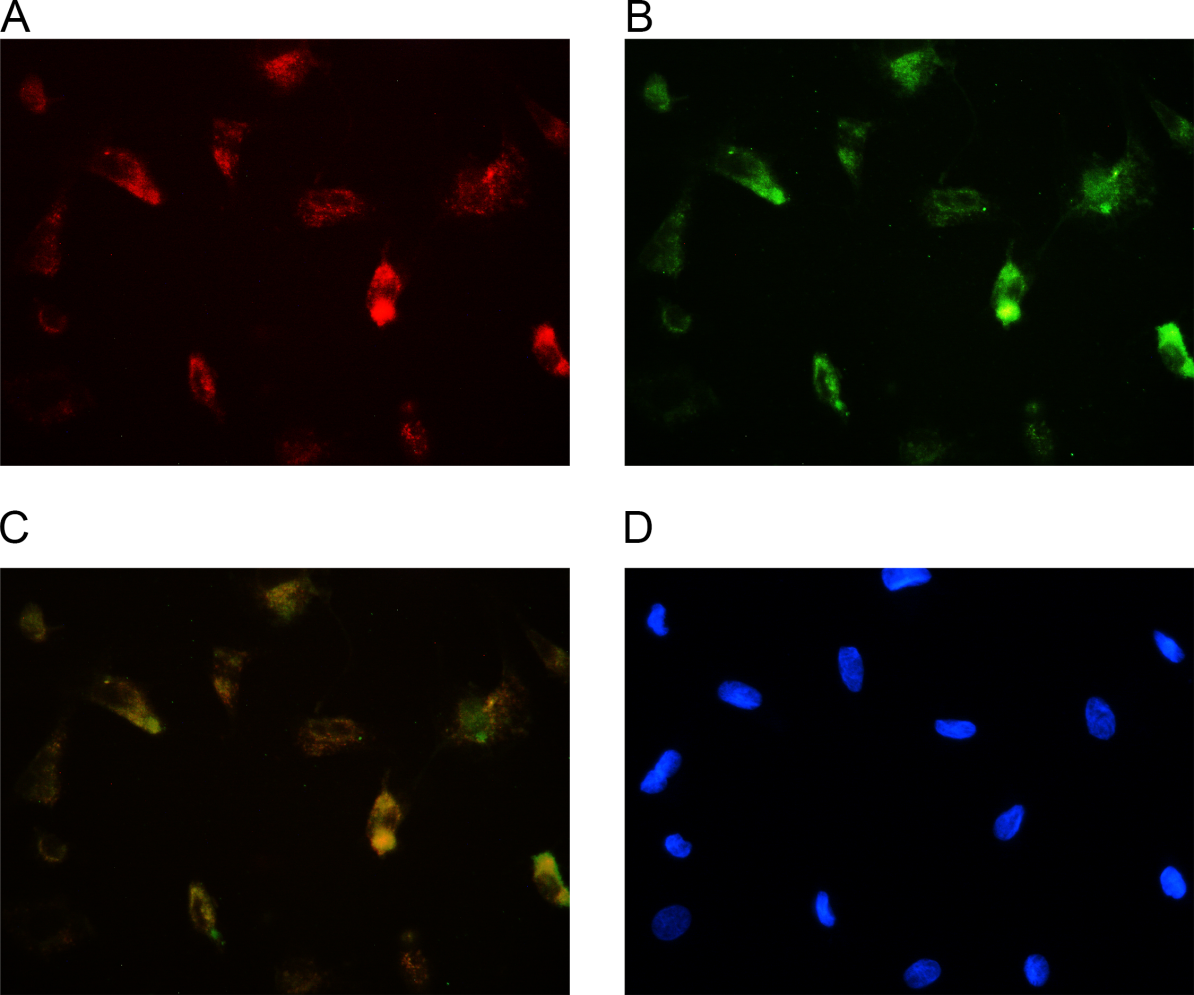
EPC characterization. Cells were positive for both DiI–acLDL uptake (A) and UEA-1 lectin binding (B) under fluorescence microscope, the markers of EPCs. C: Overlay. D: Nuclear counterstaining was performed using DAPI (blue).

**Fig 3:**
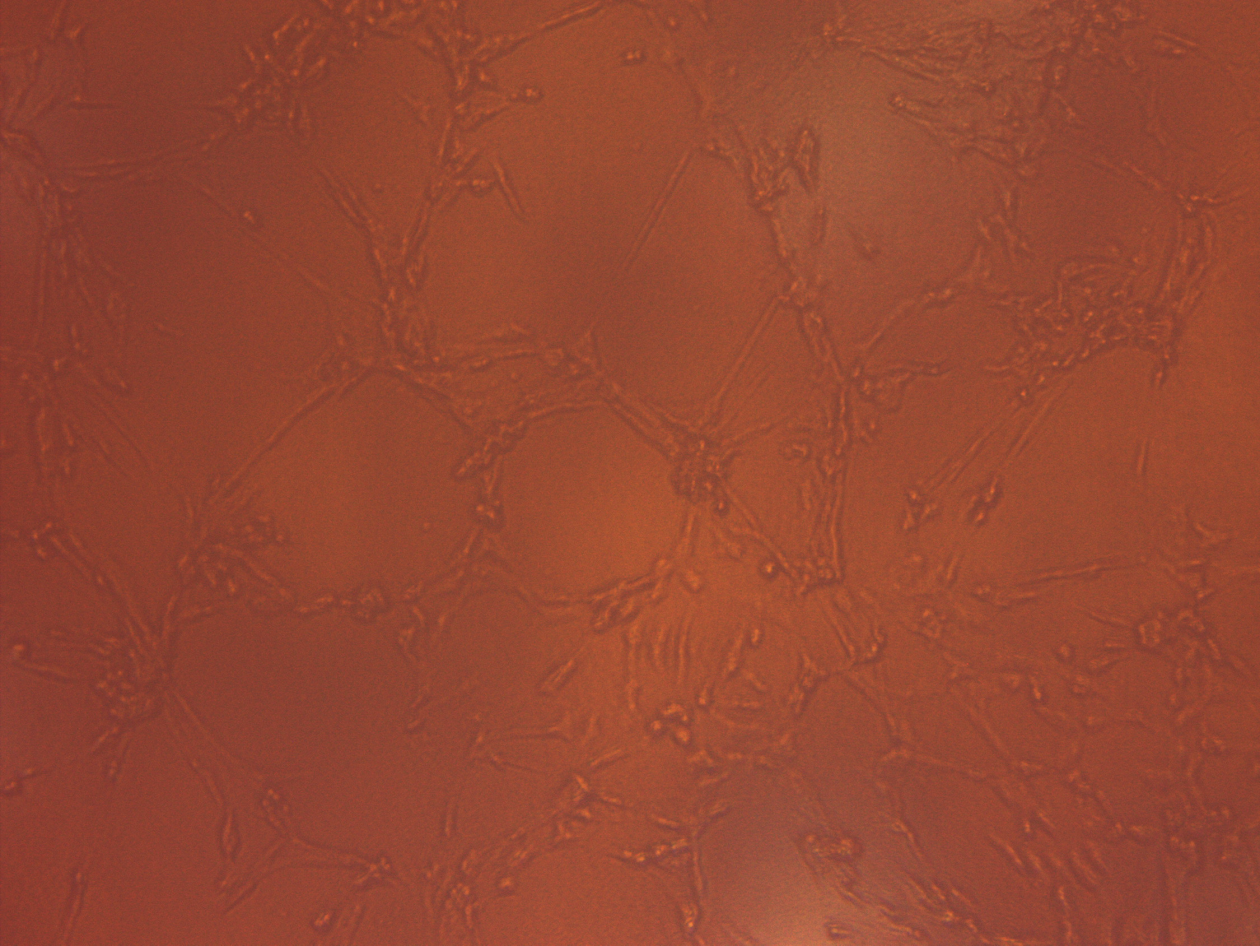
Adherent cells formed tubes in 2 days when cultured on Matrigel under an inverted light microscope.

Primary BMSCs began to adhere to the inner walls of the flask after 12 h, but cell mass still existed. At 96 h, cell adherence was obvious and the cell volume increased, with the nucleus lying in the middle of the cell. In the third generation, cells showed a typical long-spindle shape and good growth status with logarithmic growth. Some cell adherence was observed after the primary EPCs were cultured for 24 h. Nonadherent cells were gradually removed by replacing the culture medium, and the EPCs were purified. The cell volume decreased, with the shape of a short spindle or pebble. Upon sorting the cells through a flow cytometer, the following phenotypes were noted: 97.1% of BMSCs were CD29-positive and all others were CD34- and CD45-negative (Fig 1A–1C). After 1 week of culturing, EPCs were confirmed by the DiI–acLDL uptake and UEA-1 lectin binding (Fig 2A–2D), as determined by fluorescence microscopy. The capillary formation capability of the attached cells was confirmed using the Matrigel network formation assay. After 48 h, capillary-like structures appeared (Fig 3).

### Expression patterns of MCP-1 and SDF-1 in BMSCs expressing eGFP in ex vivo cultures

**Fig 4:**
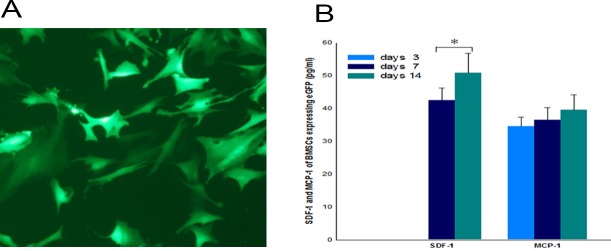
Observation and protein expression of BMSCs expressing eGFP. A: Expression of eGFP was observed in BMSCs. Selection using DMEM/F-12 culture medium containing 0.5 μg/ml puromycin yielded transduced cells with constant, stable, and bright green fluorescence after 3 weeks. B: SDF-1 and MCP-1 of BMSCs expressing eGFP by ELISA.SDF-1 and MCP-1 protein expression of BMSCs expressing eGFP in ex vivo cultures at days 3, 7, and 14. The quantity of SDF-1 and MCP-1 gradually increased with time. *p < 0.05, *n* = 4.

To focus our study on the specific homing phase, we developed an in vivo tail vein injection model, which allowed the evaluation of site-specific seeding of stem cells. BMSCs with constant, stable, and bright green fluorescence were obtained after 3 weeks (Fig 4A). ELISA results showed that the quantity of SDF-1 gradually increased with time. The secretion difference of MCP-1 at days 7 and 14 presented no statistical significance (Fig 4B).

### Partially deproteinized bones (PDPBs) in ex vivo cultures

**Fig 5:**
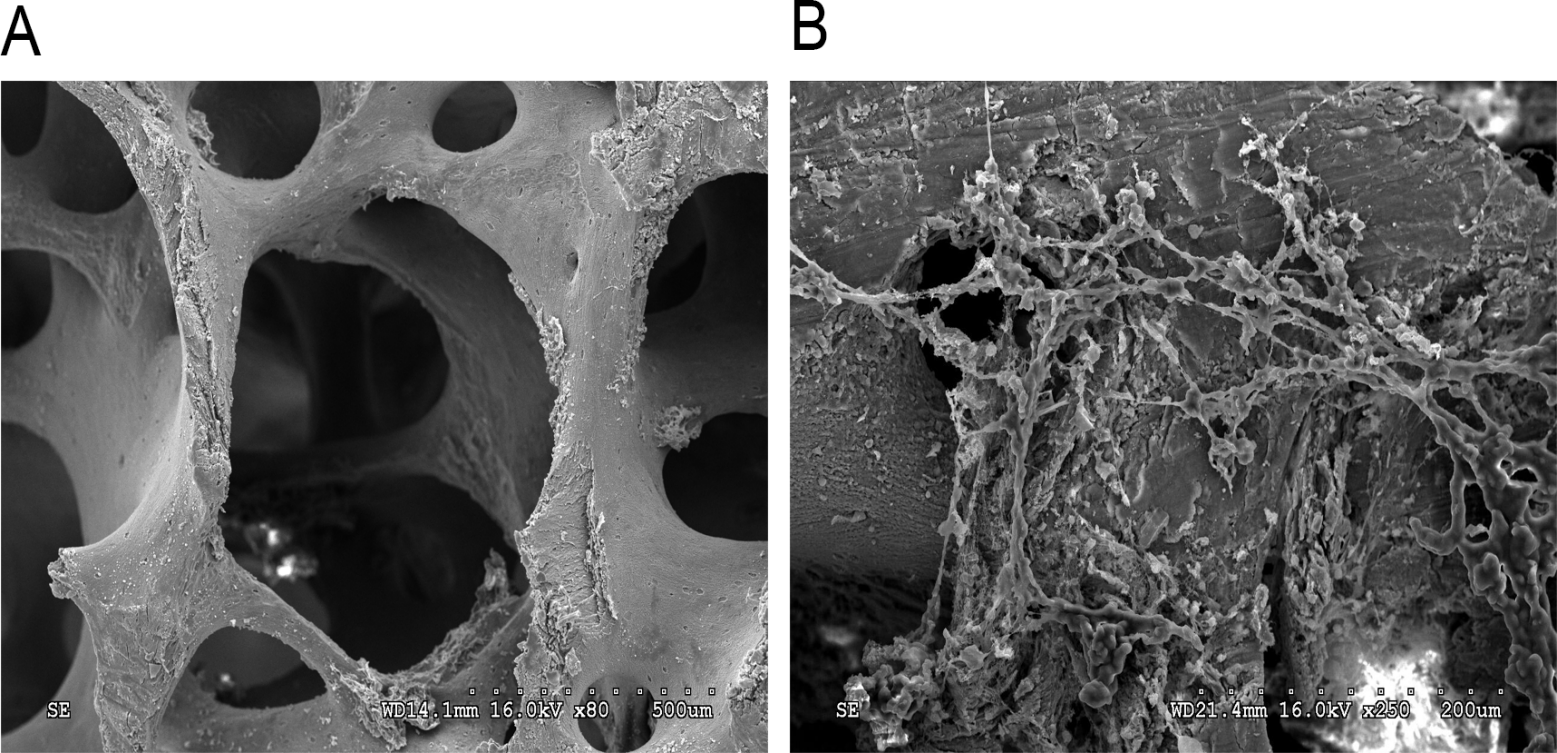
Electron microscopy images of PDPB. A: PDPB formation; interconnected pores with different sizes were observed on PDPB. B: PDPB-cell combination was cultured before implantation. Cells that adhered to the PDPB proliferated and expanded, producing large amounts of multifilament extracellular matrix deposited by the cells.

The PDPB was made largely of ivory-white color (Fig 5A). No floating attachments were found after washing the PDPB with double-distilled water. Compared with mineralized bones, the PDPB was softer, with higher plasticity and smooth tactility. As observed under a scanning electron microscope, after the cell-combined PDPB was cultured for 7 days ex vivo, confluent cells covered the surface of PDPB, and large amounts of multifilament were produced, which almost infiltrated the pores. The cells and the PDPBs combined closely (Fig 5B).

### SDF-1/CXCR4 and MCP-1/CCR2 mRNA levels

**Fig 6:**
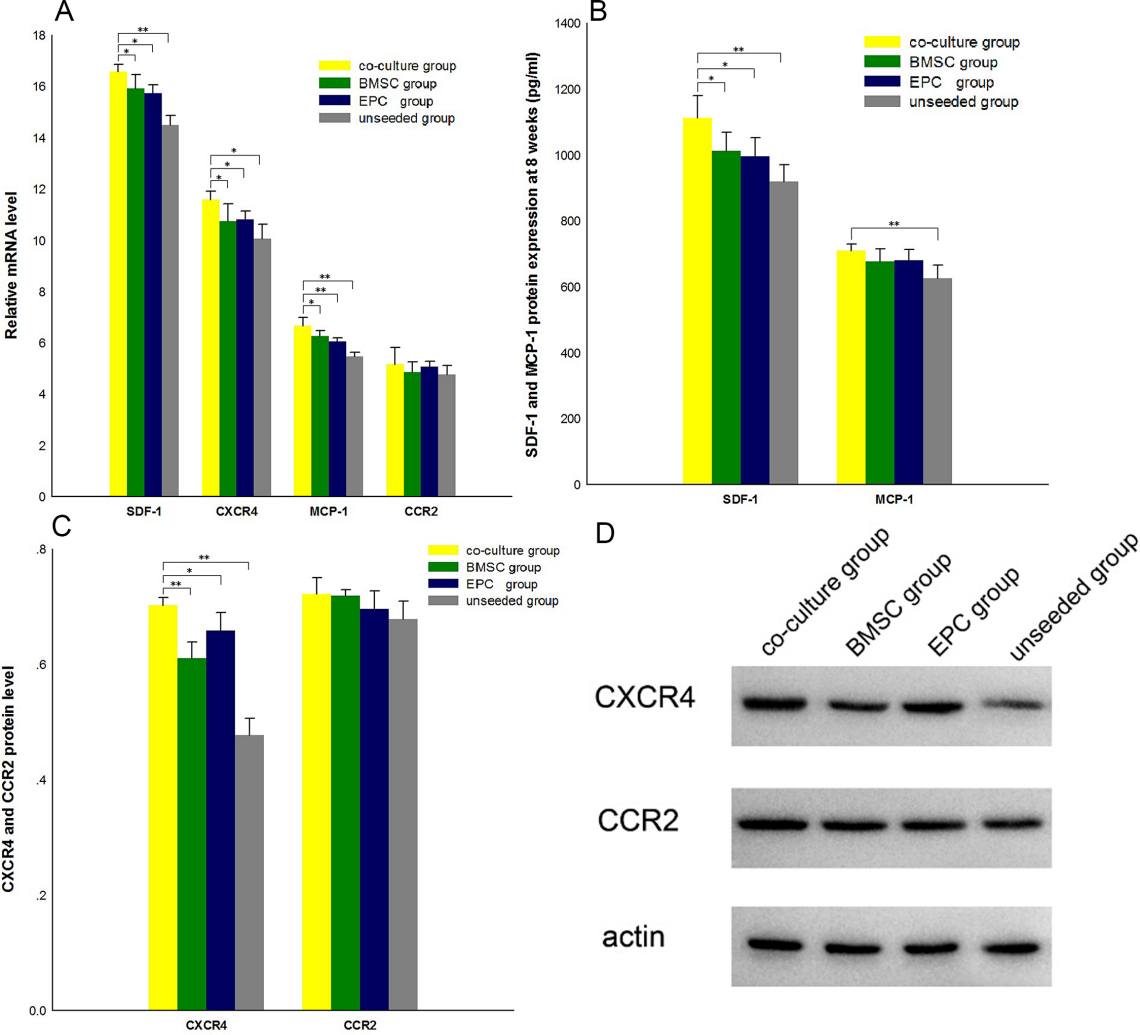
SDF-1, CXCR4, MCP-1, and CCR2 mRNA and protein levels. A: EPCs upregulated SDF-1, CXCR4, and MCP-1 mRNA levels. SDF-1, CXCR4, and MCP-1 mRNA levels in the co-culture group were higher than those in other groups. *p < 0.05, * *p < 0.01, *n* = 4. CCR2 mRNA levels exhibited no difference between groups. B: SDF-1 and MCP-1 protein levels were detected using ELISA at 8 weeks. SDF-1 level in the co-culture group was greater than that in the BMSC group, the EPC group, and the unseeded group. *p < 0.05, * *p < 0.01, *n* = 4. MCP-1 level in the co-culture group was greater than that in the unseeded group. *p < 0.05, *n* = 4. However, no significant difference in MCP-1 level was found among the other groups. C: CXCR4 and CCR2 protein levels of the four groups were analyzed by Western blot. The only change observed was upregulation of CXCR4 protein level by EPCs. *p* < 0.05, * *p < 0.01, *n* = 4. D: Western blot electrophoretogram showing CXCR4 and CCR2 protein levels.

To assess the involvement of SDF-1/CXCR4 and MCP-1/CCR2 in the homing of BMSCs promoted by EPCs, qPCR was performed to determine the relative expression levels of SDF-1/CXCR4 and MCP-1/CCR2 mRNAs in the defective tissues. Results showed that relative expression levels of SDF-1 mRNAs in the co-culture group were higher than those in the BMSC group (*p* = 0.037 < 0.05), the EPC group (*p* = 0.012 < 0.05), and the unseeded group (*p* = 0.000 < 0.01). The mRNA levels of CXCR4, the SDF-1 receptor, in the co-culture group were also higher than those in the BMSC group (*p* = 0.036 < 0.05), the EPC group (*p* = 0.046 < 0.05), and the unseeded group (*p* = 0.001 < 0.01). MCP-1 mRNAs in the co-culture group were higher than those in the BMSC group (*p* = 0.034 < 0.05), the EPC group (*p* = 0.003 < 0.01), and the unseeded group (*p* = 0.000 < 0.01). However, the mRNA levels of the MCP-1 receptor CCR2 in the co-culture group showed no significant difference compared to the BMSC group (*p* = 0.345 > 0.05), the EPC group (*p* = 0.773 > 0.05), and the unseeded group (*p* = 0.204 > 0.05) (Fig 6A). These observations indicated that the homing phenomenon may be related to the SDF-1/CXCR4 axis and MCP-1.

### Protein quantification of SDF-1/CXCR4 and MCP-1/CCR2

Based on our data on mRNA levels of SDF-1, its receptor CXCR4, and MCP-1, we deduced that the SDF-1/CXCR4 and MCP-1/CCR2 chemotactic axes were relevant for the promotion of BMSC homing. In order to find out if these mRNA levels translate to changes at the protein levels, we also determined the protein levels of the four factors. At 8 weeks, SDF-1 secretion in the co-culture group was greater than that in the BMSC group (*p* = 0.029 < 0.05), the EPC group (*p* = 0.015 < 0.05), and the unseeded group (*p* = 0.000 < 0.01). The CXCR4 protein level in the co-culture group was higher than that in the BMSC group (*p* = 0.000 < 0.001), the EPC group (*p* = 0.037 < 0.05), and the unseeded group (*p* = 0.000 < 0.001). However, MCP-1 secretion in the co-culture group was only greater than that in the unseeded group (*p* = 0.004 < 0.05) and not in the other groups (p>0.05). The CCR2 protein levels were not statistically different between the co-culture group and the other groups (*p* >0.05) (Fig 6B–6D).

### Positively stained eGFP area in the injected BMSCs

**Fig 7:**
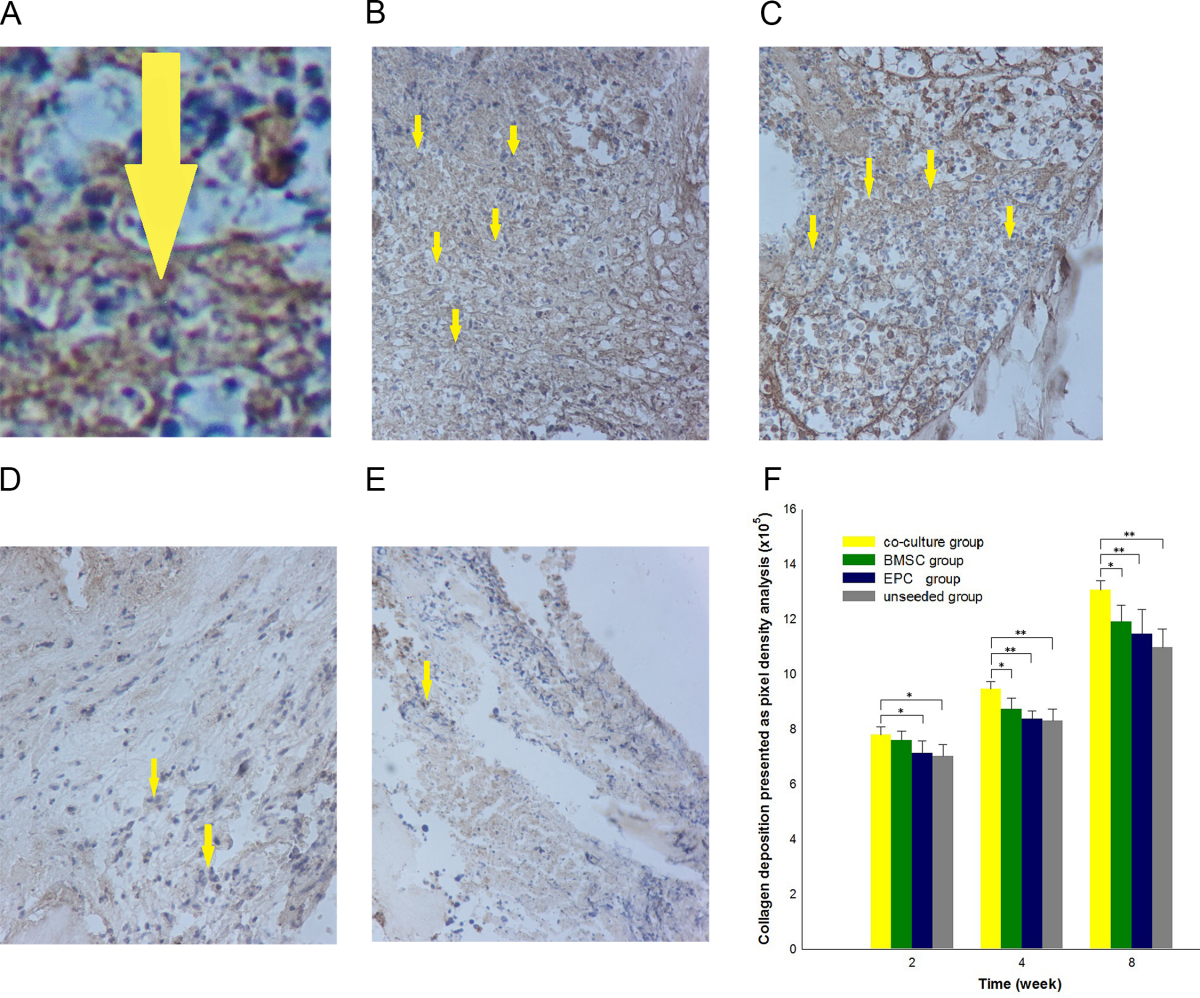
Immunohistochemical assay of eGFP-positive regions. A: Higher resolution of an example location. B–E: Representative immunohistochemical sections at 8 weeks post-surgery. F: Homing ability of BMSCs expressing eGFP was analyzed by quantifying eGFP-positive regions. No statistical differences were detected in eGFP expression among the various groups during the first 2 week (P > 0.05). After 4 weeks, expression in the experimental group was found to be significantly stronger than that in groups B, C and D. *p < 0.05, * *p < 0.01, *n* = 4.

After the animals were injected through the caudal vein with a suspension of BMSCs showing stable expression of eGFP, the BMSCs migrated to the bone defect area. Immunohistochemical assays indicated that after tail intravenous injection of BMSCs expressing eGFP, expression of eGFP could be detected in all the groups in the eighth week (Fig 7A–7E). No difference was detected in eGFP expression among these groups during the second week (*p* >0.05). However, the positive expression in co-culture group was stronger than that in the other groups after 4 weeks (*p* <0.05) (Fig 7F).

### Masson’s trichrome staining

**Fig 8:**
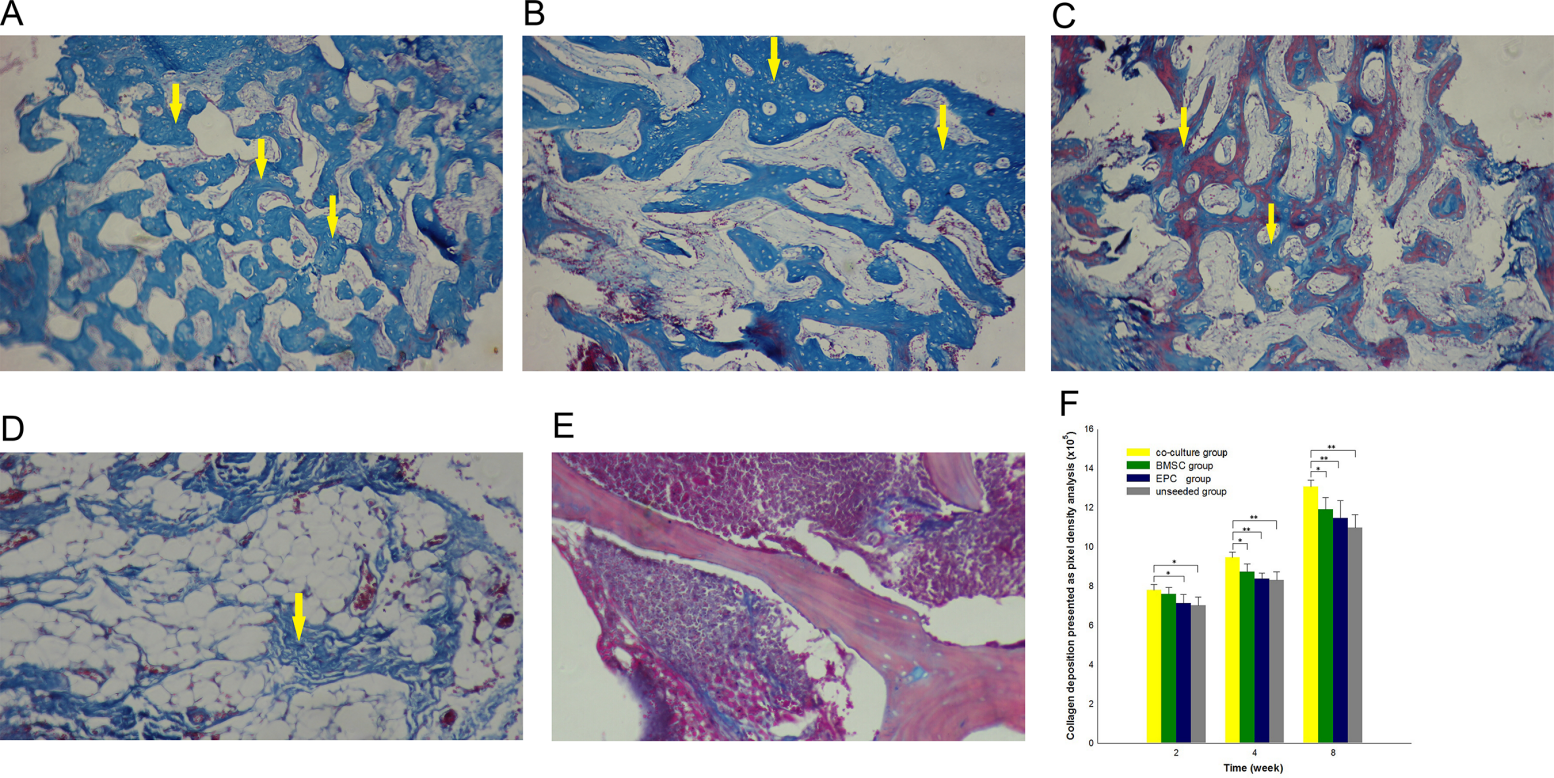
Masson’s trichrome staining. A–D: Collagen was observed in co-culture groups, the EPC group, and the unseeded group at 8 weeks. Blue staining indicates new collagen deposition and red staining indicates muscles and cytoplasm. E: A negative control image **(A**fter removing proteins, collagen was barely detected in the PDPB**)**. F: Newly formed bone in the form of collagen was quantified by pixel density analysis, and EPCs were found to promote new bone formation starting the second week. *p < 0.05, * *p < 0.01, *n* = 4.

Collagen deposition is important for the formation of new bones. After removing proteins, collagen on PDPB was barely detected. Thus, to confirm the contribution of EPCs in repairing bone defects, the osteogenic capability of the tissue-engineered bone was evaluated by quantitatively analyzing deposition of newly formed collagen using Masson’s Trichrome staining in all groups *in vivo* (Fig 8A–8E). Software analysis determined that collagen composition of the co-culture group was higher than that of the other groups at each time point (*p* < 0.05). Collagen in the co-culture group developed rapidly from the fourth week to the eighth week, whereas those in the other groups increased steadily (Fig 8F).

## Discussion

The slow pace of *in vivo* osteogenesis is a challenge in the clinical application of tissue-engineered bones for repairing bone defects. Ensuring sufficient quantity, stable adhesion, and fast proliferation of seed cells on scaffolds are important for successful transplantation in bone tissue engineering. The scaffold adhesion rate of seed cell is limited when tissue-engineered bones are implanted into the body, and immunological rejection caused by the allograft further reduces the seed cells of the scaffold. In addition, regulating homeostasis, mobilizing endogenous stem cells, and promoting homing ability of stem cells are crucial means of accelerating osteogenesis *in vivo* [25].

In the present study, PB–EPCs were used as ancillary cells to establish a co-culture system with BMSCs (target stem cells). By combining the co-culture system and the PDPB designed by the research group and implanting them into the body, we found that the eGFP-positive area of the co-culture group tissue was larger than that of the other groups after 4 weeks. Binding of SDF-1 and its receptor CXCR4 leads to activation of the SDF-1/CXCR4 axis, which plays an important role in the recruitment of BMSCs and in directed migration. Furthermore, results of our qPCR analyses revealed that the mRNA levels of SDF-1 and its receptor CXCR4 and MCP-1 were higher in the co-culture group than in the other groups, which indicated that SDF-1, CXCR4 and MCP-1 were involved in the BMSC homing process promoted by BP–EPCs.

ELISA results showed that SDF-1 in the co-culture group was significantly higher than those in the BMSC groups and the EPC group at 8 weeks after surgery. This finding indicated that co-culture of PB–EPCs and BMSCs promoted higher SDF-1 expression. The result was also consistent with CXCR4 expression in all the groups, indicating that PB–EPCs significantly increased SDF-1 /CXCR4 levels. Thus we concluded that an association between PB-EPC and BMSC recruitment mediated by the SDF-1/CXCR4 axis that can enhance repair of bone defects MCP-1 in all the groups showed no statistical difference except between co-culture groups and the unseeded group. Similarly, the CCR2 protein levels of all the groups showed no statistical difference at 8 weeks.

SDF-1/CXCR4 is an important homing axis of BMSCs that also plays a crucial role in homing and migration of hematopoietic stem cells [26, 27] and mobilization of bone marrow-derived osteoblast cells [28]. Fujio M et al. [29] used a mouse fracture model to demonstrate that SDF-1 enhances osteogenesis-mediated skeletal tissue regeneration by recruiting endothelial precursors. Ryu et al. [30] showed that migration of human umbilical cord blood mesenchymal stem cells (hUCB–MSCs) was also mediated by SDF-1/CXCR4, and that the Akt, ERK, and p38 signaling pathways were involved in hUCB–MSC migration by SDF-1. SDF-1/CXCR4 mobilizes calcium, decreases cyclic AMP within cells, and activates multiple signal transduction pathways, including PI3K, phospholipase C-c/protein kinase C, and the MAP kinases ERK1/2 [31, 32]. Interestingly, Wang J [33] found that MAPK/ERK was not required for SDF-1-mediated migration of progenitor cells. Thus, the signal transduction pathways involved in SDF-1/CXCR4-mediated cell migration appears to be cell type-specific. Cheng M et al. [34] identified the Src family protein kinases as critical downstream effectors of SDF-1/CXCR4 signaling that play an essential role in the chemotactic response of bone marrow progenitor cells. Except for SDF-1/CXCR4, stem cell homing is also related to HGF/c-met [35] and VLA-4/VCAM-1 [36] receptor–ligand axes.

SDF-1, which binds its receptor CXCR4 and leads to the formation of SDF-1/CXCR4 axis, is important for recruitment of BMSCs and directed migration [37]. SDF-1 is also vital in MSCs differentiating into cartilages and bones [38,19]. The effects of SDF-1 on potentiating the migration of host MSCs and enhancing healing of osteochondral defects has been demonstrated previously [38]. In a mouse fracture model, SDF-1 was shown to promote bone regeneration by recruiting MSCs [19] to the injured bone. Hosogane et al. showed that blocking the SDF-1 signaling pathway inhibited BMP2-induced osteogenic differentiation, indicating that the SDF-1 signaling pathway is essential to the osteogenic process [39]. Zhu et al. also demonstrated that blocking SDF-1/CXCR4 signaling strongly inhibited BMP2-induced osteogenic differentiation of ST2 BMSCs, and the interaction between SDF-1 and BMP2 signaling was mediated via intracellular Smad and MAPK activation [40]. Results of Masson’s trichrome staining in our study showed that PB–EPCs of the co-culture group caused significantly greater bone formation than the other groups without PB–EPCs. This result is consistent with our findings on changes in SDF-1 expression. Thus, we conclude that EPCs might act on BMSCs through SDF-1, which also promotes osteogenesis of BMSCs. Overall, these results are consistent with and supportive of the findings of previous studies [19,39,40]. However, this study has some limitations. For instance, the eGFP-positive areas do not only reflect the quantity of homing BMSCs, but also the proliferation of eGFP-expressing BMSCs. Further investigation is needed to determine the signaling pathways activated when EPCs mediate homing of BMSCs.

In summary, EPCs are closely related to homing, osteogenesis, and angiogenesis of BMSCs. The multiple functions of EPCs make them potential seed cells for the construction of tissue-engineered bones. Our findings suggest that the gene expression patterns related to BMSC homing change during co-culture with EPCs. Hence, targeted gene modification in BMSCs is expected to be a direct and effective measure for promoting BMSC homing to injured or defective sites. This assumption provides new ideas for the acceleration of stem cell homing *in vivo*.
